# A histone deacetylase inhibitor enhances rice immunity by derepressing the expression of defense-related genes

**DOI:** 10.3389/fpls.2022.1041095

**Published:** 2022-11-02

**Authors:** Yan Xu, Yuanxin Miao, Botao Cai, Qingping Yi, Xuejun Tian, Qihai Wang, Dan Ma, Qiong Luo, Feng Tan, Yongfeng Hu

**Affiliations:** ^1^ Hubei Engineering Research Center for Specialty Flowers Biological Breeding/College of Bioengineering, Jingchu University of Technology, Jingmen, China; ^2^ Center for Science Popularization Jingmen, Science and Technology Museum, Jingmen, China; ^3^ State Key Laboratory for Conservation and Utilization of Bio-Resources in Yunnan/Ministry of Education Key Laboratory of Agricultural Biodiversity for Plant Disease Management, Yunnan Agricultural University, Kunming, China; ^4^ Department of Biochemistry and Molecular Biology, College of Life Sciences, Nanjing Agricultural University, Nanjing, China; ^5^ Key Laboratory of Three Gorges Regional Plant Genetics and Germplasm Enhancement, Biotechnology Research Center, China Three Gorges University, Yichang, China

**Keywords:** HDAC inhibitors, rice blast, histone acetylation, defense-related genes, rice

## Abstract

Histone deacetylase (HDAC) inhibitors (HDACis) have been widely used in plants to investigate the role of histone acetylation, particularly the function of HDACs, in the regulation of development and stress response. However, how histone acetylation is involved in rice (*Oryza sativa L.*) disease resistance has hardly been studied. In this paper, four HDACis including Sodium butyrate (NaBT), Suberoylanilide Hydroxamic Acid (SAHA), LBH-589 and Trichostatin A (TSA) were used to treat rice seedlings at different concentrations before inoculation of *Magnaporthe oryzae*. We found that only 10mM NaBT treatment can significantly enhanced rice blast resistance. However, treatment of the four HDACis all increased global histone acetylation but at different sites, suggesting that the inhibition selectivity of these HDACis is different. Notably, the global H3K9ac level was dramatically elevated after both NaBT and LBH589 treatment although LBH589 could not enhance rice blast resistance. This indicates that the HDACs they inhibit target different genes. In accordance with the phenotype, transcriptomic analysis showed that many defense-related genes were up-regulated by NaBT treatment. Up-regulation of the four genes *bsr-d1*, *PR10B*, *OsNAC4*, *OsKS4* were confirmed by RT-qPCR. ChIP-qPCR results revealed that H3K9ac level on these genes was increased after NaBT treatment, suggesting that these defense-related genes were repressed by HDACs. In addition, by promoter motif analysis of the genes that induced by both NaBT treatment and rice blast infection, we found that the motifs bound by ERF and AHL transcription factors (TFs) were the most abundant, which demonstrates that ERF and AHL proteins may act as the candidate TFs that recruit HDACs to defense-related genes to repress their expression when plants are not infected by rice blast.

## Introduction

Rice (*Oryza sativa L*.) is one of the most important food crops and the main food source for more than 50% people in the world. Rice blast is the major diseases that affect the yield and quality of rice, and considered as the most serious threat to global rice production. The annual yield loss caused by rice blast can reach 10%~30% of the total rice yield (Skamnioti and Gurr, 2009; Zhou, 2016). At present, the commonly used methods to control rice blast mainly include cultivating resistant rice varieties, chemical agents and improved cultivation measures. Repeated and excessive use of fungicides to control plant diseases has lead to the serious global environment pollution. It is of great significance to reveal the molecular mechanism of rice blast resistance and cultivate resistant varieties for the safe production of rice. Large quantities of defense-related genes in rice have been identified. Those defense-related genes encode various recognition receptors, signal transduction proteins and transcription factors (Chujo et al., 2013; Lee et al., 2016; Li et al., 2017; Li et al., 2019b; Yin et al., 2021). In addition, epigenetic regulators also play important roles in disease resistance *via* regulating expression of defense-related genes (Zhu et al., 2016).

Histone acetylation, which is deposited by histone acetyltransferases (HATs) and removed by histone deacetylase (HDACs), is positively engaged in the regulation of gene expression. The HDACs can be classified into three families: Reduced Potassium Dependency 3 (RDP3)/Histone Deacetylase 1 (HDA1), Silent Information Regulator 2 (SIR2), and the plant-specific Histone Deacetylase 2 (HD2) (Pandey et al., 2002). The plant RPD3/HDA1 family is divided into three classes (I, II and IV) based on their homology to yeast HDACs (Ueda et al., 2017). In plants, the involvement of HDACs in the immune response has extensively studied. In Arabidopsis, HDA19 acts as a positive regulator of plant immunity, as overexpression of *HDA19* enhances plant resistance to the necrotrophic pathogen *Alternaria brassicicola* (Zhou et al., 2005). In *HDA19*-overexpression plants, expression of genes related to jasmonic acid and ethylene signaling is increased (Zhou et al., 2005). Besides, HDA19 inhibits transcription activation activity of WRKY38 and WRKY62 that function as negative regulators of plant immunity (Kim et al., 2008). However, later study revealed that *HDA19* negatively regulated plant resistance to *Pseudomonas syringae* by repressing *PR1* and *PR2* (Choi et al., 2012 *)*. So the role of *HDA19* in the regulation of plant immunity remains controversial. HDA9 and HOS15 form the complex HDA9-HOS15 to inhibit the expression of *NLR* genes by reducing H3K9ac and negatively regulate the immunity to *Pseudomonas syringae* pv *tomato* DC3000 (*Pst* DC3000) (Yang et al., 2020). The loss-of-function of *HOS15* or *HDA9* results in the increased expression of one third of 207 *NLR* genes and the consequent enhancement of resistance to pathogen infection. HDA6 inhibits pathogen-responsive genes by removing H3 acetylation at corresponding loci, thus negatively regulating plant immunity (Wang et al., 2017). In wheat, TaHOS15 serves as an adaptor protein to recruit TaHDA6 into the chromatin of defense-related genes (such as *TaPR1*, *TaPR2*, *TaPR5* and *TaWRKY45*, etc.), which together inhibit the acetylation of H3 and H4 at these genes (Liu et al., 2019). Down-regulation of *TaHOS15* or *TaHDA6* enhances defense-related genes expression under the infection of *blumeria graministhe*, which leads to the decreased susceptibility to *Blumeria graminis* f.sp. *tritici* (*Bgt*). TaHDT701 also can form HDAC complex with TaHDA6 and TaHOS15 to repress the expression of defense-related genes (Zhi et al., 2020). In rice, The function of rice HDT701, which is responsible for H4 deacetylation, in innate immunity has been characterized (Ding et al., 2012). Transgenic rice overexpressing *HDT701* is more sensitive to rice blast and bacterial leaf blight. On the contrary, *HDT701* RNAi plants exhibit enhanced resistance to rice blast and bacterial leaf blight. After treated with pathogen-related molecular pattern effectors, *HDT701* RNAi plants produce more reactive oxygen species and accumulate more transcripts of pattern recognition receptor genes *FLS2*, *CEBiP* and *SGT1*. In addition, HDT701 directly binds to defense-related genes *MAPK6* and *WRKY53* to inhibit their expression. These results demonstrate that HDT701 is a negative regulator of rice immune response.

HDAC inhibitors (HDACis) can specifically inhibit the activity of HDAC, and are widely used to clarify the function of these enzymes. A variety of HDACis with different selectivity to HDAC proteins have been developed (Waterborg, 2011; Patanun et al., 2016; Ueda et al., 2017; He et al., 2020). The research on application of HDACis in plants are also increasing. For example, Ky-2 and SAHA treatments enhance salt tolerance in Arabidopsis and cassava respectively (Patanun et al., 2016; Sako et al., 2016). Seven other HDACis screened by Ueda et al. can also induce salinity stress tolerance in Arabidopsis (Ueda et al., 2017). HDACis have been applied in rice to study the function of HDAC in root development and callus formation (Chung et al., 2009; Zhang et al., 2020). In this paper, we selected four HDACis to treat rice seedlings before inoculating *M. oryzae.* The plants treated with 10mM NaBT displayed enhanced resistance to rice blast, while those treated with the other HDACis did not. Global H3K9ac level was increased after NaBT treatment, suggesting the inhibitory effect of NaBT on HDAC activity. Furthermore, a number of defense-related genes were activated and H3K9ac levels on these genes were increase by NaBT treatment. These results indicate that NaBT enhances rice blast resistance by inhibiting the activity of HDACs, thereby increasing H3K9ac level and activating defense-related genes.

## Materials and methods

### Plant materials and growth conditions

Rice (*Oryza sativa ssp japonica* cv Hwayoung) plants were germinated and grown in a growth room kept at 26°C and 70% relative humidity with a 12-h light/12-h dark cycle. The 7-day-old rice seedlings were treated with different concentrations of four HDACis, NaBT (1 mM, 5 mM, 10 mM, 20 mM, Wako Pure Chemical Industries, 193-01522), SAHA (100 uM, 300 uM, 500 uM, 1 mM, Selleck Chemicals LLC, S1047), LBH-589 (100 uM, 300 uM, 500 uM, 1 mM, Selleck Chemicals LLC, S1030) and Trichostatin A (500 uM, 1 mM, Sigma-Aldrich, T1952), respectively. HDACis with the same concentration were added when the rice hydroponic medium or nutrient solution was changed every week. DMSO-treated plants act as a control. After three weeks of 10 mM NaBT treatment of rice seedlings (28-day-old), the third leaves of NaBT-treated plants and the control were harvested at 12, 24 and 48h thereafter (**Figure S1**). Ten individual plants were harvested for each sample. The harvested samples were immediately put into liquid nitrogen for quickly freezing, and stored in a -70°C refrigerator for transcriptome sequencing, RT-qPCR, ChIP-qPCR and measurement of defense-related enzyme activity.

### Pathogen infection assay and disease evaluation

The 28-day-old rice seedlings treated with HDACis were inoculated by spraying with *M. oryzae* JC2 spore suspension (2.0×10^5^ spores/mL), and the DMSO-treated plants were used as the control (**Figure S1**). Five days after inoculation, the incidence of disease was counted (**Figure S1**). At least 80 leaves were used for counting for each treatment. The disease grade was identified according to the evaluation standard of rice blast resistance grade (Li et al., 2021).

### Western blot analysis

Rice seedlings were treated with 10 mM NaBT, 1 mM SAHA, 1 mM LBH-589, 1 mM TSA as described above (**Figure S1**). Leaves were harvested and nuclear proteins were extracted by using extraction buffer I(0.4 M Sucrose, 10 mM pH 8.0Tris-HCl, 10 mM MgCl2, 5 mM β-ME, 0.1 mM PMSF, 1×complete protease inhibitors), extraction buffer II (0.25 M Sucrose, 10 mM pH 8.0 Tris-HCl, 10 mM MgCl_2_, 10% Triton X-100, 5 mM β-ME, 0.1 mM PMSF, 1×complete protease inhibitors) and extraction buffer III (1.7 M Sucrose, 10 mM Tris-HCl(pH 8.0), 2 mM MgCl_2_, 0.15% Triton X-100, 5 mM β-ME, 0.1 mM PMSF, 1×complete protease inhibitors) successively. Histone proteins were separated by 15% SDS-PAGE followed by immunoblotting using specific primary antibodies: H3 (Abcam, ab1791), H4(Millipore, 06–598), H3K9ac (Millipore, 07-352), H3K27ac (Millipore, 07-360), H4K5ac (Millipore, 07-327), H4K16ac (Millipore, 07-329) and secondary antibody (Earthox, E03120-01). Chemiluminescent detection were performed with ECL reagent according to the manufacturer’s protocol (Millipore, Cat.No.WBKLS0050). ImageJ software was used to quantify the band intensities.

### RNA-seq and data analysis

Total RNA of Rice seedlings without roots was extracted using TRNzol Universal Reagent (TIANGEN). The RNA quality for RNA-seq and RT-qPCR was examined using a Bioanalyzer 2100 (Agilent). RNA-seq libraries were generated using the UltraTM RNA Library Preparation Kit (NEB, USA)for Illumina. A total of 18 libraries were sequenced on an Illumina platform and 150 bp paired-end read were generated. To obtain the RNA-seq data, Trimmomatic (version 0.33) was used to removing contaminations and low-quality reads (Xu et al., 2022). And clean reads were mapped to the reference genome of rice (MSU7.0) by Hisat2 v2.2.0 (Xu et al., 2022). To normalize counts and analyze the difference of RNA expression level, the R package of DESeq2 (v4.0.3) were used with adjusted P-value<0.05 and |log2 (Fold change)| ≥1 (Xu et al., 2022). KEGG analyses were conducted using the online site [DAVID Functional Annotation Bioinformatics Microarray Analysis (ncifcrf.gov)].

### Chromatin immunoprecipitation

Chromatin Immunoprecipitation (ChIP) experiment was conducted as described in Hu et al. (2020). 2g of rice seedling without roots was cross-linked with 1% (v/v) formaldehyde before being used to isolate nuclei and chromatin. The chromatin was broken into 200 to 250bp fragments by sonication and immunoprecipitated with H3K9ac antibody. Dyna-beads Protein G (Invitrogen, 10003D) was used to harvest the immunocomplex. After extensive washing, immunoprecipitated chromatin was de-cross-linked to release the DNA for ChIP-qPCR.

### RT-qPCR and ChIP-qPCR

According to the manufacturer’s protocol, cDNAs were synthesized by reverse transcription with HiScript II QRT SuperMix for qPCR (+gDNA wiper) (Vazyme). RT-qPCR and ChIP-qPCR were performed using SuperReal PreMix Plus (SYBR Green) (TIANGEN) on an ABI System (Stepone). The PCR reactions were performed under the following conditions: preincubation at 95 °C for 15 min, then 40 cycles of 95 °C for 10 s, and 60 °C for 1min (Xu et al., 2022). In addition, the expression levels were calculated using the 2^−ΔΔCt^ method for each sample (Xu et al, 2022). For RT-qPCR the *Actin* gene was used as an internal control. The primers used in this study are listed in **Table S1**.

### Measurement of SOD, POD, CAT and PAL activity

After sampling, according to the instructions of the detection kit (Total superoxide dismutase (T-SOD) test kit A001-1-2, Phenylalanine ammonia lyase (PAL) test kit A137-1-1, Peroxidase (POD) assay kit A084-3 -1, Catalase (CAT) assay kit A007-1-1, all from Nanjing Jiancheng Bioengineering Institute), first add the extraction solution and liquid nitrogen for grinding, and then perform the detection according to the operation steps.

## Results

### NaBT treatment enhances rice resistance to *M. oryzae*

**Figure 1:**
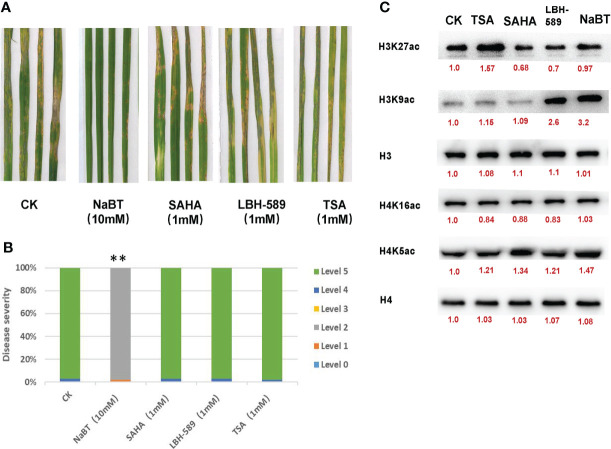
The effect of HDAC inhibitors treatment on rice blast resistance and histone acetylation in rice seedlings. **(A)** Leaf phenotypes after *M. oryzae* inoculation. Seven-day-old rice seedlings were treated with 10 mM NaBT, 1 mM SAHA, 1 mM LBH-589, 1 mM TSA. DMSO treatment serves as a control. After three weeks treatment the seedlings were inoculated with *M. oryzae.* Disease severity were investigated five days after inoculation. **(B)** Estimation of the disease grade after *M. oryzae* inoculation in **(A)** (**P< 0.01). **(C)** Immunoblot analysis of histone acetylation in rice seedlings treated with 10mM NaBT, 1 mM SAHA,1 mM LBH-589, 1 mM TSA. ImageJ software was used to quantify the band intensities (red number).

In order to analyze the role of histone acetylation in the regulation of defense response in rice, four HDACis including NaBT, SAHA, LBH-589 and TSA were used to treat rice seedlings at different concentrations in the liquid culture medium one week after germination. The plants treated with DMSO were used as a control. The fresh liquid culture medium with HDACis was replaced every week. After three weeks treatment, we found that the shoot growth was not obviously affected and the plants were inoculated with *M. oryzae.* The results indicate that only treatment of NaBT at the concentration greater than 10mM could significantly enhance rice resistance to *M. oryzae* (**Figure 1A**; **Figure S2A**). The resistance grade was determined as grade 2 intermediate resistance (**Figure 1B**; **Figure S2B**). The plants treated with the other HDACis at various concentrations displayed similar resistance grade with the control (determined as grade 5 susceptible) (**Figure 1B**; **Figure S2B**). This suggests that only NaBT could activate rice defense response possibly by increasing histone acetylation at defense genes.

In response to pathogen infection, the activity of several defense-related enzymes are activated such as Superoxide dismutase (SOD), Peroxidase (POD), Catalase (CAT) responsible for scavenging reactive oxygen species (ROS) and Phenylalanine ammonia lyase (PAL) that is the key enzyme for phenylpropanoid biosynthesis (Appu et al., 2021; Song et al., 2022). To determine whether NabT-induced disease resistance involves these enzymes, we detected the activity of them after NaBT treatment. We found that the activity of different enzymes responded to NaBT in a different manner (**Figure S3**). In response to NaBT treatment, PAL activity was gradually increased after NaBT treatment, CAT activity was first increased and then decreased, whereas POD activity was deceased at early stage and then increased, SOD activity was however inhibited at three time points. These results indicate that the activity of the defense-related enzymes was affected by NaBT treatment, evidencing that defense response is activated after rice seedling is treated with NaBT.

### Global H3K9ac is increased in rice after NaBT treatment

Several studies have shown that treatment of NaBT, TSA, SAHA can increase the acetylation level of histone H3 and H4 (Chua et al., 2003; Ueda et al., 2017; He et al., 2020), and treatment of LBH-589 can increase the acetylation level of histone H3 (Ueda et al., 2017). In order to investigate the effect of these four HDACis on the level of histone acetylation in rice, we treated rice seedlings with 10 mM NaBT, 1 mM SAHA, 1 mM LBH-589, and 1 mM TSA using the same method as described above. After three weeks treatment, the nucleoproteins of rice leaves were extracted. Western Blot was performed by using antibodies against H3K27ac, H3K9ac, H4K5ac, H4K16ac and internal reference H3 and H4. The results showed that TSA treatment slightly increased H4K5ac level and dramatically increase H3K27ac level (**Figure 1C**; **Figure S4**). SAHA treatment remarkably increased H4K5ac level. H3K9ac level significantly rose after LBH-589 and NaBT treatment (**Figure 1C**; **Figure S4**). These data demonstrate that different HDAC inhibitors may inhibit different HDACs leading to the increased histone acetylation at different sites, and NaBT mainly inhibits the HDACs responsible for H3K9 deacetylation in rice. Previously we reported that genome-wide H3K9ac was related to the activation of defense-related genes (Xu et al., 2022), supporting that inhibition of H3K9 deacetylation by NaBT treatment possibly causes derepression of defense-related genes.

### RNA-seq analysis reveals up-regulation of defense-related genes by NaBT treatment

**Figure 2:**
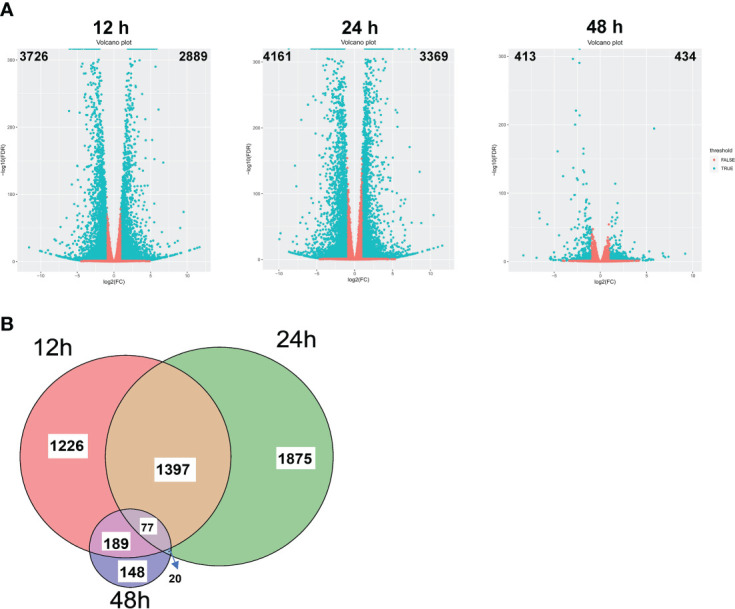
Transcriptomic changes induced by NaBT treatment. **(A)** Volcano plot of differential gene expression at 12, 24 and 48h induced by 10 mM NaBT. Red dots indicate genes with unchanged expression, and blue dots indicate differentially expressed genes (DEGs). Overlapping of up-DEGs **(B)** at 12, 24 and 48h treated with 10mM NaBT is presented by venn diagram.

To understand the underlying mechanism of enhanced blast resistance in rice seedlings under NaBT treatment, samples were harvested at 12h, 24h and 48h after rice seedlings were treated with 10 mM NaBT. Total RNA was extracted and high-throughput sequencing was performed. More than 40M clean reads were obtained for each sample and were mapped to the rice reference genome (MSU7.0), and the mapping rate all exceeded 96% (**Table S2**). Differentially expressed genes (DEGs) between NaBT treatment and control were analyzed at the three time points respectively based on the transcriptome data. The results showed that 2889, 3369 and 434 genes were up-regulated (up-DEGs, log2 (Fold change)≥1, FDR<0.05) at 12, 24 and 48h, respectively, while 3726, 4161 and 413 genes were down-regulated (down-DEGs, log2 (Fold change) ≤-1, FDR<0.05) at 12, 24 and 48 hours, respectively (**Figure 2A**). These data demonstrated that NaBT dramatically affected transcriptional programme within 24h, and the effect was attenuated at 48h after treatment. We analyzed H3K9ac enrichment on the up-DEGs (a total of 4932 genes at the three time points) by using ChIP-seq data we had published previously (Xu et al., 2022). The samples used for RNA-seq and ChIP-seq experiment were 28-day-old seedlings inoculated with *M. oryzae* (Xu et al., 2022), which are consistent with the materials used in this study. We found that 3459 genes were marked with H3K9ac, which comprised about 70% of all the up-DEGs. These genes might be the potential targets regulated by histone deacetylases, whose activity is inhibited by NaBT. Unexpectedly, more down-DEGs than up-DEGs were observed (**Figure 2A**), which was not consistent with the predicted consequence after HDAC activity was inhibited by NaBT, since HDACs are mainly involved in gene repression. It is possible that many transcriptional repressors may be induced after NaBT treatment, thus resulting in the down-regulation of large amounts of genes. In addition, we found that most of up-DEGs were transiently up-regulated at different time points except that 77 genes were induced at all three time points (**Figure 2B**; **Table S3**). These genes continuously up-regulated by increased histone acetylation might serve as candidates for responding to developmental or environmental stimuli epigenetically. However, among these 77 genes no defense-related genes have been annotated. This indicates that defense-related genes can only be activated temporarily after the activity of histone deacetylases is inhibited.

**Figure 3:**
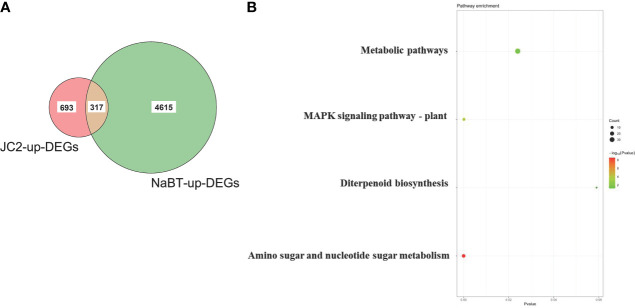
Identification of genes induced by both rice blast infection and NaBT treatment. **(A)** The number of genes induced by rice blast infection (JC2) and NaBT treatment is presented by venn diagram. **(B)** The Kyoto Encyclopedia of Genes and Genomes (KEGG) analysis of 317 up-DEGs identified in **(A)**.

**Figure 4:**
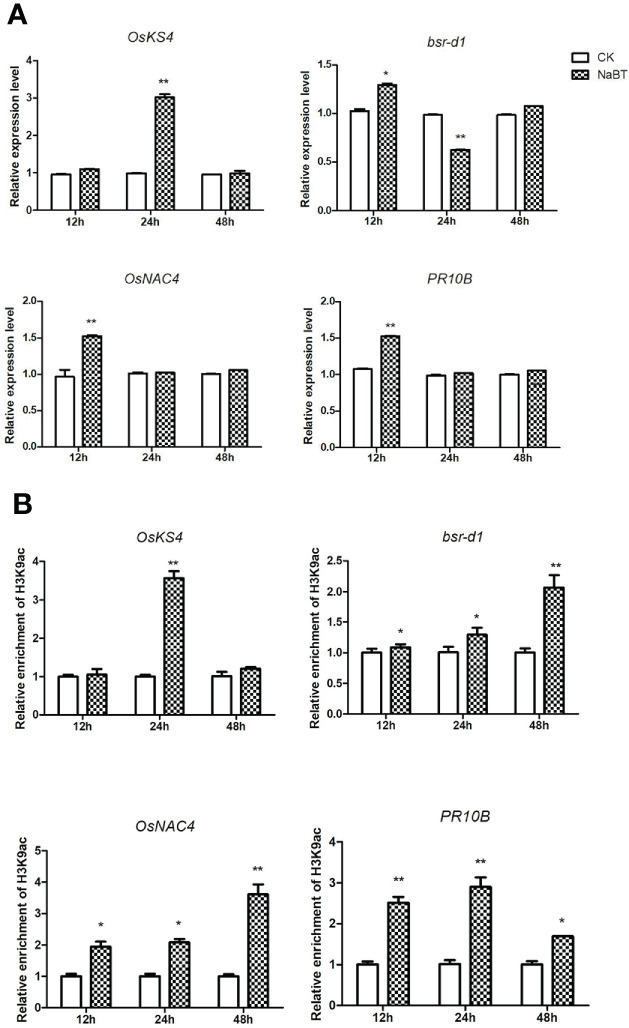
Validation of four defense-related gene expression **(A)** and H3K9ac enrichment **(B)** on these genes responding to NaBT treatment by RT-qPCR and ChIP-qPCR. The *actin* gene was used as internal control for RT-qPCR. The input was used as internal control for ChIP -qPCR. Three independent biological repeats were performed for each experiment. (*P< 0.05; **P< 0.01).

To further identify the defense-related genes regulated by histone deacetylases, we combined the RNA-seq data in this study and in our previously published paper where transcriptomic change at 12h, 24h, 48h after inoculation of *M. oryzae* has been analyzed (Xu et al., 2022). Totally 1110 genes were induced by rice blast infection at the three time points. We found that about one third of these rice blast-induced genes (317 genes) were up-regulated by NaBT treatment (**Figure 3A**; **Table S4**). KEGG enrichment pathway analysis revealed that 317 genes were concentrated in metabolic pathways, amino sugar and nucleotide sugar metabolism, plant MAPK signaling pathway, and diterpenoid biosynthesis (**Figure 3B**). In addition, 27 of 317 up-DEGs were annotated as defense-related genes (**Table 1**), which means the up-regulated expression of these 27 defense-related genes is likely to be both induced by *M. oryzae* and regulated by histone acetylation. To verify the up-regulation of these 27 defense-related up-DEGs by NaBT treatment, we selected four genes *bsr-d1*, *PR10B*, *OsNAC4*, *OsKS4* for RT-qPCR. The results indicated that the expression levels of *OsKS4* at 24 h, *OsNAC4* at 12 h, *bsr-d1* at 12 h, *PR10B* at 24 h were significantly increased (**Figure 4A**), which is consistent with the RNA-seq results.

**Table 1:** List of 27 defense-related genes up-regulated by inoculation of *M. oryzae* (JC2) and NaBT treatment.

Gene locus No.	Gene name	Log2(FC) induced by JC2			Log2(FC) induced by NaBT		
		12h	24h	48h	12h	24h	48h
LOC_Os01g21120	*OsERF68*	0.25	1.36	0.28	-1.4	2.13	-0.26
LOC_Os01g28500	*OsPR1#012*	-0.12	1.74	2.46	1.18	2.81	-0.07
LOC_Os01g43550	*OsWRKY12*	1.05	0.4	3.13	2.52	-0.65	0.24
LOC_Os01g60020	*OsNAC4*	0.59	0.84	1.12	1.57	0.75	0.23
LOC_Os01g71670	*OsPR2*	0.28	2.03	1.8	1.62	3.49	-0.19
LOC_Os02g32610	*OsCTR2*	0.55	1.2	0.74	2.88	0.21	0.23
LOC_Os02g36140	*OsKS7*	0.77	1.66	2.39	-0.29	1.68	-1.61
LOC_Os03g32230	*bsr-d1*	0.69	0.9	1.35	1.15	-1.44	-0.72
LOC_Os03g49260	*OsLOX3*	0.91	0.95	1.22	4.6	1.84	-0.92
LOC_Os03g52860	*LOX-L2*	1.92	1.68	0.55	2.46	3.22	-0.9
LOC_Os04g10060	*OsKS4*	2.08	3.15	3.34	-0.21	2.91	0.13
LOC_Os04g41620	*Cht4*	0.13	3.29	3.24	0.76	3.33	0.23
LOC_Os04g41680	*Cht5*	-0.14	2.45	1.99	-0.5	2.18	-0.22
LOC_Os05g33130	*Cht2*	1.4	3.33	5.35	3.5	4.43	-0.03
LOC_Os05g33140	*Cht9*	0.92	2.24	0.28	1.16	2.89	-0.81
LOC_Os06g44010	*OsWRKY28*	1.67	1.02	0.4	1.24	0.69	-0.72
LOC_Os06g51050	*Cht3*	2.72	4.25	3.7	0.56	2.33	0.3
LOC_Os06g51060	*Cht1*	0.88	1.01	1.24	0.23	1.19	-0.83
LOC_Os07g48730	*OsRLCK241*	0.62	0.53	1.07	0.87	1.76	-0.63
LOC_Os09g25070	*OsWRKY62*	1.41	2.36	1.06	0.01	2.72	-2.74
LOC_Os10g02070	*poxA*	0.32	0.06	1.65	1.46	0.38	0.31
LOC_Os10g11500	*OsPR1#101*	2.72	3.44	5.61	-0.35	4.47	0.04
LOC_Os11g37960	*OsPR4b*	1.11	1.12	1.54	1.46	0.65	-0.34
LOC_Os11g37970	*OsPR4*	0.58	2.76	1.75	0.31	1.96	-0.24
LOC_Os11g47580	*RIXI*	-0.27	3.85	2.79	2.79	5	-0.13
LOC_Os12g36850	*PR10B*	0.75	0.95	2.14	-0.45	1.3	-1.91
LOC_Os12g36860	*RPR10*	0.78	0.89	2.23	0.21	2.04	-2.32

The global increment of histone acetylation induced by NaBT treatment was expected to result from either inhibition of HDAC activity or mis-regulation of HAT and HDAC genes. To verify the hypothesis, we obtained the expression value of these genes from our RNA-seq data and visualized the fold-change in response to NaBT treatment by heat maps. We found that three HAT genes (*HAG703*, *HAC701* and *HAC704*) and four HDAC genes (*HDA704*, *HDA709*, *HDA710*, and *HDA716*) were activated, and one HAT gene (*HAG704*) and two HDAC genes (*HDA714* and *OsSRT2*) were repressed by NaBT treatment (**Figure S5A** and **S5B**). Thus, based on these results we proposed that transcriptional activation of HAT genes or repression of HDAC genes induced by NaBT could possibly contribute to the increase of histone acetylation.

### H3K9ac on four NaBT-induced defense-related genes is increased after NaBT treatment

To confirm whether the four defense-related genes *OsKS4*, *bsr-d1*, *OsNAC4* and *PR10B* were regulated by histone acetylation, ChIP-qPCR were performed using the samples harvested at 12h, 24h and 48h after NaBT treatment. The H3K9ac level on *bsr-d1*, *OsNAC4* and *PR10B* was significantly increased at 12, 24 and 48h although the expression of these genes are increased only at 12h and decreased to the level comparable with the control (**Figure 4B**). We speculate that these genes may be activated by NaBT treatment at early stage, but their transcriptional repressors are also up-regulated giving rise to the repression of the three defense-related genes at later stage possibly independent of histone deacetylation. The H3K9ac level on *OsKS4* was also significantly increased at 24h (**Figure 4B**), which is in accordance with the induced expression of *OsKS4* at 24h after NaBT treatment (**Figure 4A**). The results indicate that up-regulation of the four defense-related genes *OsKS4*, *bsr-d1*, *OsNAC4* and *PR10B* by NaBT treatment all involves histone acetylation. As these genes are also induced by rice blast infection, we would like to learn whether induction of these genes by rice blast infection necessitate histone acetylation. We used IGV software to visualize the data of H3K9ac enrichment before and after inoculation of *M. oryzae* we obtained previously (Xu et al., 2022). It was shown that H3K9ac was enriched on *bsr-d1* and *OsNAC4* but not on *OsKS4* and *PR10B* (**Figure S6A**). Besides, quantitative analysis suggested that H3K9ac on all four genes were not significantly increased after inoculation of *M. oryzae* (**Figure S6B**), which indicates that induction of these defense-related genes in response to rice blast infection does not involve H3K9ac increase.

### ERF and AHL TF-binding Cis-elements are enriched in the promoter of NaBT-induced defense genes

**Figure 5:**
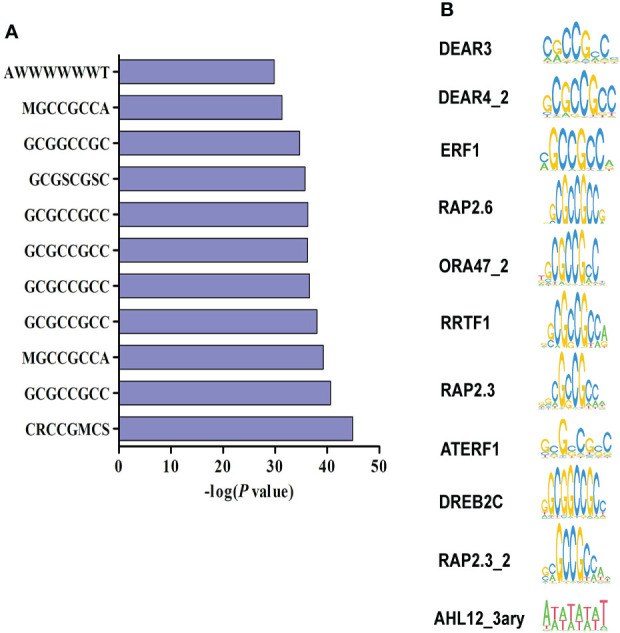
Identification of transcription factor binding motifs related to the repression of defense-related genes by HDAC. **(A)** Promoter motif enrichment analysis of 317 genes up-regulated by both rice blast infection and NaBT treatment. W represents for A or T, S represents for C or G, R represents for A or G, M represents for A or C. **(B)** The motifs enriched in the promoters of 317 up-DEGs.

HDACs could be recruited to target genes by various TFs to exert histone deacetylation (Li et al., 2019a; Xu et al., 2022). To identify TFs that may be responsible for the recruitment of HDACs to *M. oryzae*-inducible genes, we performed TF-binding motif analysis in the promoters of 317 genes that both induced by *M. oryzae* and up-regulated by NaBT treatment. The results showed that most of identified motifs were GCC-box-containing cis-elements including DEAR3, DEAR4_2, ERF1, RAP2.6, ORA47_2, RRTF1, RAP2.3, ATERF1, DREB2C, and RAP2.3_2 (**Figures 5A, B**). These motifs are recognized by ERF family TFs, suggesting that recruitment of HDAC to defense-related genes are mainly mediated by ERF TFs. Interestingly, we have previously reported that ERF TFs may also recruit HATs to defense-related genes for their induction (Xu et al., 2022). We also revealed that ERF genes could either activated or repressed by rice blast infection (Xu et al., 2022). This indicates that different ERF proteins are responsible for recruiting HATs and HDACs respectively. In addition, the “AWWWWWWT” motif, designated as AHL12_3ary and bound by the AHL family proteins, were also identified. Expression analysis showed that two AHL genes (*AHL4* and *AHL9*) were down-regulated and five AHL genes (*AHL11*, *AHL15*, *AHL17*, *AHL19*, *AHL20*) were up-regulated by inoculation of *M. oryzae* (**Figure S7**). This demonstrates that AHL genes are possibly involved in defense response in rice.

## Discussion

HDACis can be divided into three classes based on their target selectivity, which has been studied in human (Ueda et al., 2017). Class I and Class II HDACis inhibit the activity of Class I and Class II HDACs of RPD3/HDA1 family, while Class III HDACis, also coined Pan-HDACi, inhibit the activity of the whole family HDACs. However, treatment of HDACis leads to increased histone acetylation at multiple sites in various plant species. For example, TSA treatment increases H4ac, H3K9ac, and H4K5ac levels in maize (Hu et al., 2011), H3ac and H4ac levels in tobacco (Chua et al., 2003), H3ac, H4ac, H3K14ac, H3K9ac, and H4K5ac levels in Arabidopsis (Mengel et al., 2017). SAHA treatment increases H3ac and H4ac levels in cassava (Patanun et al., 2016), H3K9ac and H4K5ac levels in cotton (He et al., 2020). LBH-589 and NaBT treatment increases H3ac levels in Arabidopsis (Ueda et al., 2017). NaBT treatment increases H3ac and H4ac levels in tobacco (Chua et al., 2003). In this study, we used TSA, SAHA, LBH-589 and NaBT to treat rice seedlings and detected changes of H3K9ac, H3K27ac, H4K5ac and H4K16ac levels by Western blot. We found that the treatment of different HDACis induced elevated histone acetylation level at different sites despite that TSA, LBH-589 and NaBT are classified as Pan-HDACi in human (**Figure 1C**). This suggests that the inhibition selectivity of HDACi in plants may differ from that in animals. In addition, H3K9ac levels can be enhanced by TSA treatment in maize and Arabidopsis but not significantly in rice reveled in this study. Instead, H3K27ac level was dramatically elevated after TSA treatment, which is consistent with the result reported by Zhang et al. (Zhang et al., 2020), suggesting that inhibitory effects of TSA on HDACs varies in different plant species. We speculate that the genome-wide increment of histone acetylation level induced by treatment of HDACi could be the consequence of inhibition of HDAC activity or mis-regulation of HAT or HDAC genes. Indeed, we found that several HAT and HDAC genes expression was affected by NaBT treatment (**Figures S3A, B**), indicating that these genes themselves are regulated by histone acetylation. However, it has been revealed that accumulation of HDAC1 transcripts was decreased by NaBT treatment even in HDAC1-overexpression rice plants (Chung et al., 2009). This demonstrates that NaBT may promote degradation of HDAC1 mRNA.

The distinct inhibitory effects of HDACis could also be reflected by their roles in development and stress tolerance. It has been reported that TSA can promote wheat embryo regeneration and inhibit spruce pollen tube elongation and rice callus formation (Cui et al., 2015; Bie et al., 2020; Zhang et al., 2020), enhance drought resistance of peanut cold tolerance of Arabidopsis (Song et al., 2017; Zhang et al., 2018). SAHA treatment improves salt tolerance in cassava and cotton (Patanun et al., 2016; He et al., 2020). LBH-589 treatment also improves salt tolerance in Arabidopsis (Ueda et al., 2017). NaBT was found to inhibit root growth in a dose-dependent manner in rice (Chung et al., 2009). However, the effect of HDACi treatment on plant biotic stress tolerance has never been reported before. In this study, NaBT, SAHA, TSA and LBH-589 were used to treat rice seedlings at different concentrations. Only 10 mM NaBT could enhance the rice blast resistance of rice seedlings even though NaBT and LBH-589 can both induce notable elevation of H3K9ac level (**Figures 1A-C**). We suspect that NaBT and LBH-589 inhibit the activity of different HDACs target different sets of genes. RNA-seq analysis indicated that many defense-related genes induced by rice blast infection were up-regulated by NaBT treatment. Among them, several genes have been unveiled to be involved in blast resistance such as *bsr-d1*, *PR10B*, *OsNAC4*, *OsKS4*, which were confirmed to be regulated by NaBT by RT-qPCR (**Figure 4A**). We found that H3K9ac level on these genes was increased after HDAC activity was inhibited by NaBT treatment but was not increased after rice blast infection (**Figures 4B** and **S4**), suggesting that induction of these genes in response to biotic stress does not require the increase of H3K9ac on the genes. However, inhibition of HDAC activity is indeed related to disease resistance. In Arabidopsis, S-nitrosylation of HDAC promoted by NO inhibits HDAC activity, consequently leading to global increase of H3 and H4 acetylation and activation of stress genes (Mengel et al., 2017). It has been proved that NO production can be stimulated by both SA and TSA treatment, indicating that inhibition of HDAC by NO may serve as one of the pathway to activate immune response.

In plants, recruitment of HDAC by TFs is an important way for HDAC targeting downstream genes (Liu et al., 2014). To identify possible TFs responsible for recruiting HDAC to defense-related genes, we analyzed the common motifs in promoter of the genes that were up-regulated by NaBT treatment and inoculation of *M. oryzae*. The motifs bound by ERF and AHL proteins were determined to be the most abundant (**Figures 5A, B**). The direct interaction of HDAC with both ERF and AHL proteins have been unraveled before (Deng et al., 2022; Yun et al., 2012; Han et al., 2016; Hu et al., 2022). Besides, it has been revealed that overexpression of several *AHL* genes suppressed PAMP-induced *NHO1* and *FRK1* expression in Arabidopsis (Lu et al., 2010). The negative role of *ERF* genes in plant immunity has also been reported (Huang et al., 2016). Therefore, it is speculated that ERF and AHL proteins may recruit HDAC to defense-related genes to repress their expression under normal conditions. After pathogen infection, the expression of these TF genes is repressed and the recruitment of HDAC is blocked leading to increased histone acetylation and gene activation. Indeed, we found some *ERF* and *AHL* genes were down-regulated in response to rice blast infection (Xu et al., 2022, **Figure S5**). In consistent with this hypothesis, HDA19 directly represses *PR1* and *PR2* expression by deacetylate histones at the promoter (Choi et al., 2012). Loss-of-function of *HDA19* enhances resistance to *P. syringae* (Choi et al., 2012). HDA6 and HDA9 also negatively regulate the immunity in Arabidopsis (Wang et al., 2017; Yang et al., 2020). It is of great interest to identify HDAC-interacting TFs involved in regulating plant immunity.

## Conclusion and perspectives

Collectively, in this study we found a HDAC inhibitor, NaBT, can significantly enhance rice blast resistance by activating defense-related genes with increased histone H3K9ac. The results not only prove the importance of RPD3/HDA1 family HDAC in immune response in rice, which has never been reported before, but also provide the evidence for the potential use of the HDAC inhibitors as agricultural chemicals to improve rice disease resistance. However, application of the other HDACis that also increased global histone acetylation could not enhance rice blast resistance, demonstrating that not all the HDACs are involved in rice disease resistance. Functional analysis of HDACs is required to find which HDACs are responsible for rice blast resistance.

## Data availability statement

The data presented in the study are deposited in the NCBI repository, accession number PRJNA879115.

## Author contributions

YX: Investigation; Methodology; Data curation. YM: Data curation; Formal analysis. BC: Data curation. QY: Formal analysis. XT: Data curation. QW: Formal analysis. DM: Formal analysis. QL: Writing - review and editing. Supervision; FT: Writing - review and editing. Supervision; YH: Conceptualization; Funding acquisition; Writing - original draft; Writing - review and editing. All authors contributed to the article and approved the submitted version.

## Funding

This work was supported by the Opening Project of Hubei Engineering Research Center for Specialty Flowers Biological Breeding (2022ZD005) and Natural Science Foundation of Jiangsu Province (BK20190535)

## Conflict of interest

The authors declare that the research was conducted in the absence of any commercial or financial relationships that could be construed as a potential conflict of interest.

## Publisher’s note

All claims expressed in this article are solely those of the authors and do not necessarily represent those of their affiliated organizations, or those of the publisher, the editors and the reviewers. Any product that may be evaluated in this article, or claim that may be made by its manufacturer, is not guaranteed or endorsed by the publisher.

## Supplementary material

Supplementary Figure 1 The process of NaBT treatment and sample collection in this study.

Supplementary Figure 2 Test of rice blast resistance in rice seedlings treated with HDACi at other concentrations. **(A)** Leaf phenotypes after *M. oryzae* inoculation. Seven-day-old rice seedlings were treated with 1 mM NaBT, 5 mM NaBT, 20 mM NaBT, 100 uM SAHA, 300 uM SAHA, 500 uM SAHA, 100 uM LBH-589, 300 uM LBH-589, 500 uM LBH-589, 500 uM TSA. DMSO treatment serves as a control. After three weeks treatment the seedlings were inoculated with *M. oryzae.* Disease severity were investigated five days after inoculation. **(B)** Estimation of the disease grade after *M. oryzae* inoculation in **(A)** (**, P< 0.01).

Supplementary Figure 3 The activity of defense-related enzymes change in response to NaBT treatment.

Supplementary Figure 4 Immunoblot analysis of histone acetylation in rice seedlings treated with 10mM NaBT, 1 mM SAHA,1 mM LBH-589, 1 mM TSA (Repeat of ). ImageJ software was used to quantify the band intensities (red number).

Supplementary Figure 5 Changes of HAT **(A)** and HDAC **(B)** gene expression in response to NaBT treatment.

Supplementary Figure 6 H3K9ac level on four defense-related genes responding to *M. oryzae* inoculation.

Supplementary Figure 7 Changes of AHL family gene expression in response to *M. oryzae* inoculation.
