# Supplementary figures and images for: A histone deacetylase inhibitor enhances rice immunity by derepressing the expression of defense-related genes
Source: Front Plant Sci. 2022 Nov 2;13:1041095. doi: 10.3389/fpls.2022.1041095 (PMC9667192; doi:10.3389/fpls.2022.1041095)

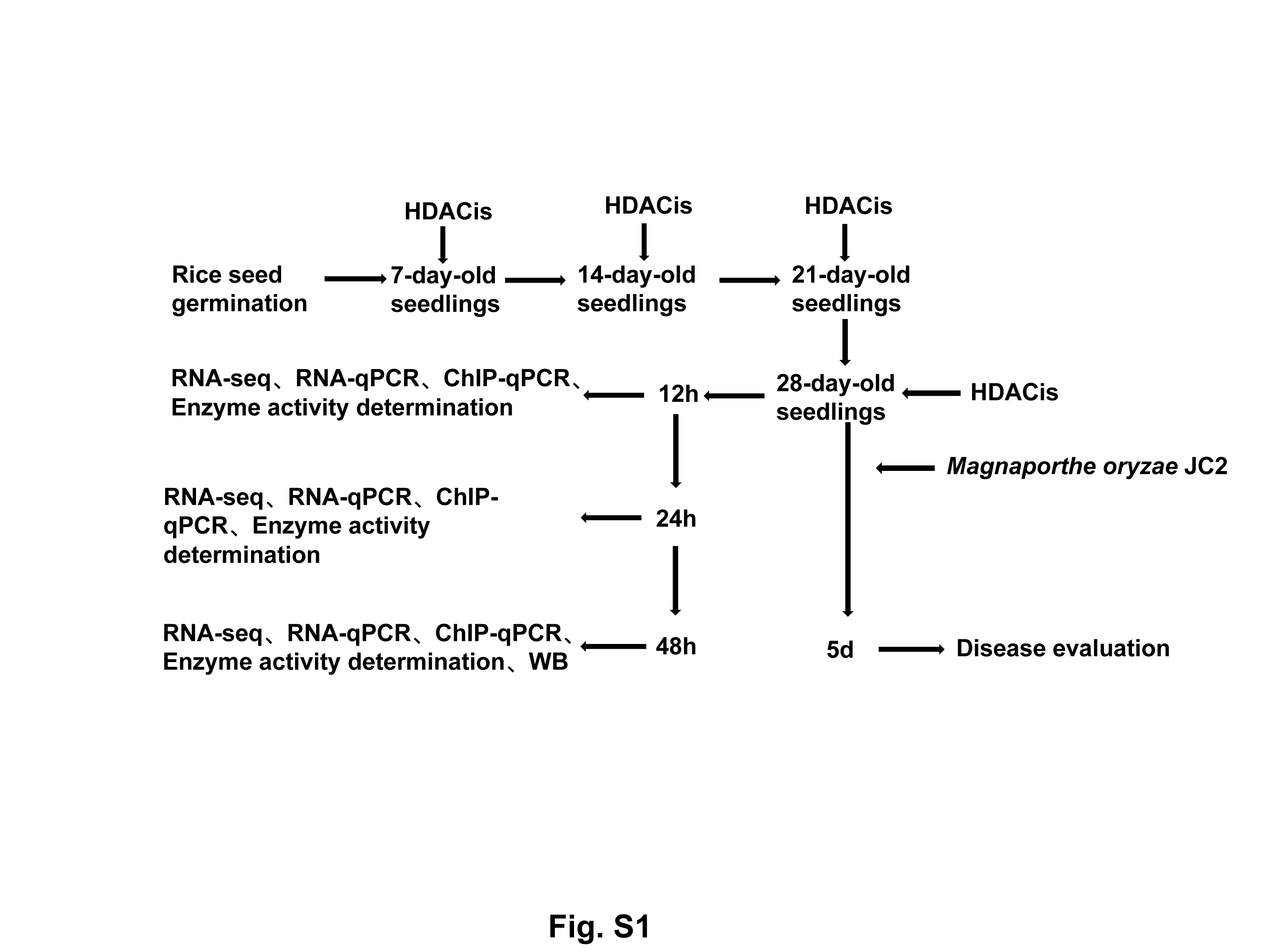

Supplement: Supplementary Figure 1 — The process of NaBT treatment and sample collection in this study. [file DataSheet_1.zip › Fig S1.jpg]

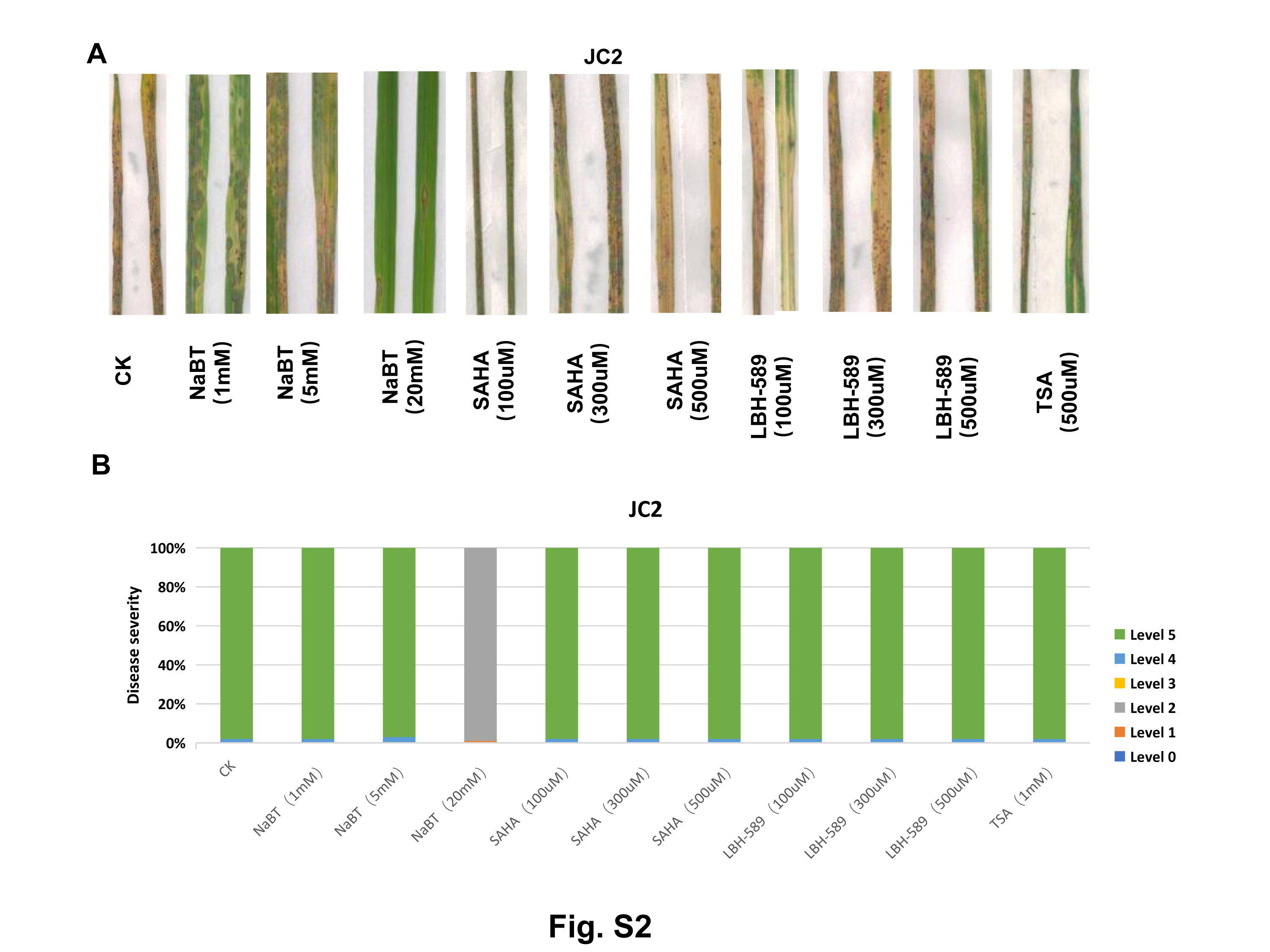

Supplement: Supplementary Figure 1 — The process of NaBT treatment and sample collection in this study. [file DataSheet_1.zip › Fig S2.jpg]

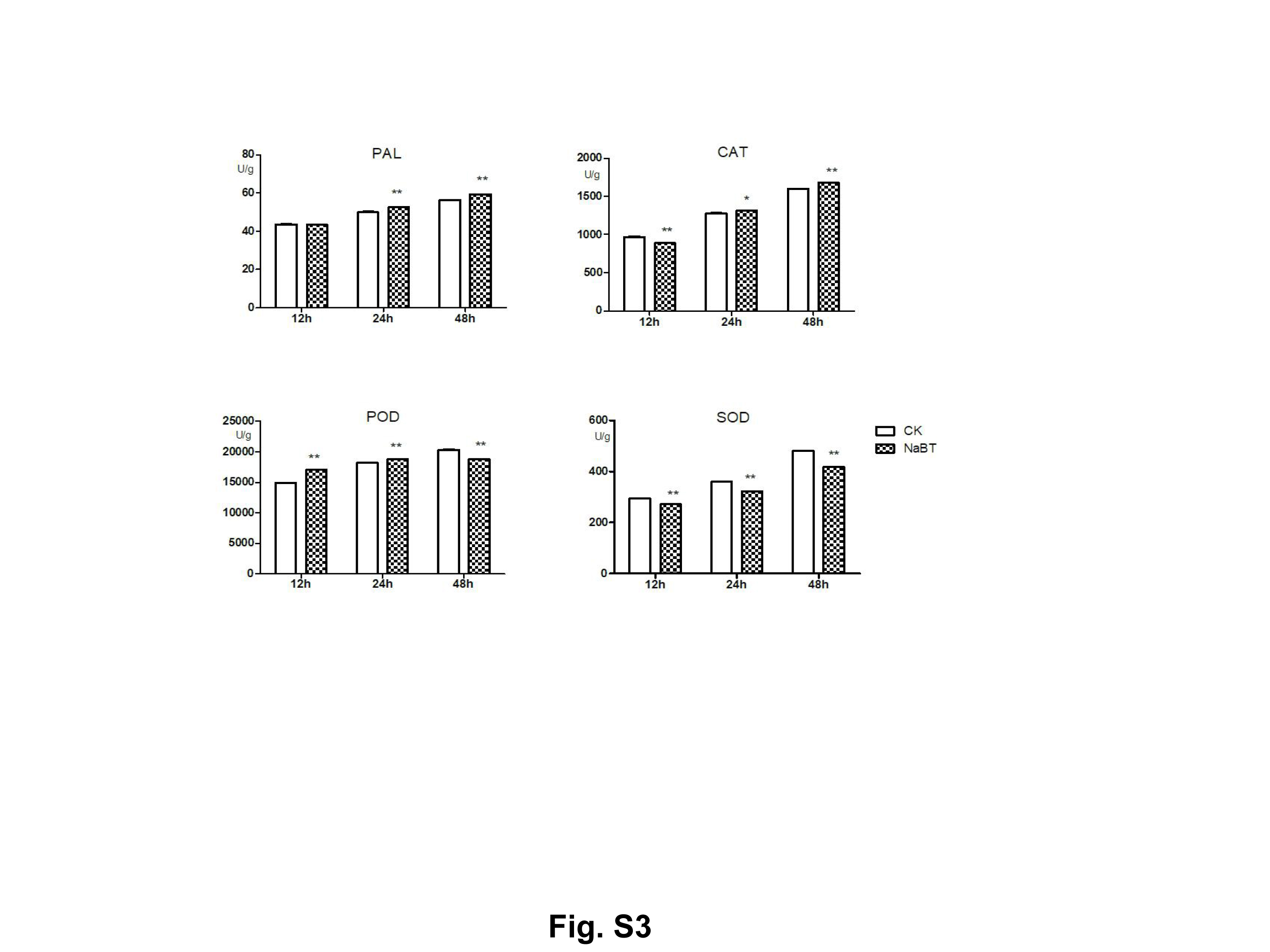

Supplement: Supplementary Figure 1 — The process of NaBT treatment and sample collection in this study. [file DataSheet_1.zip › Fig S3.jpg]

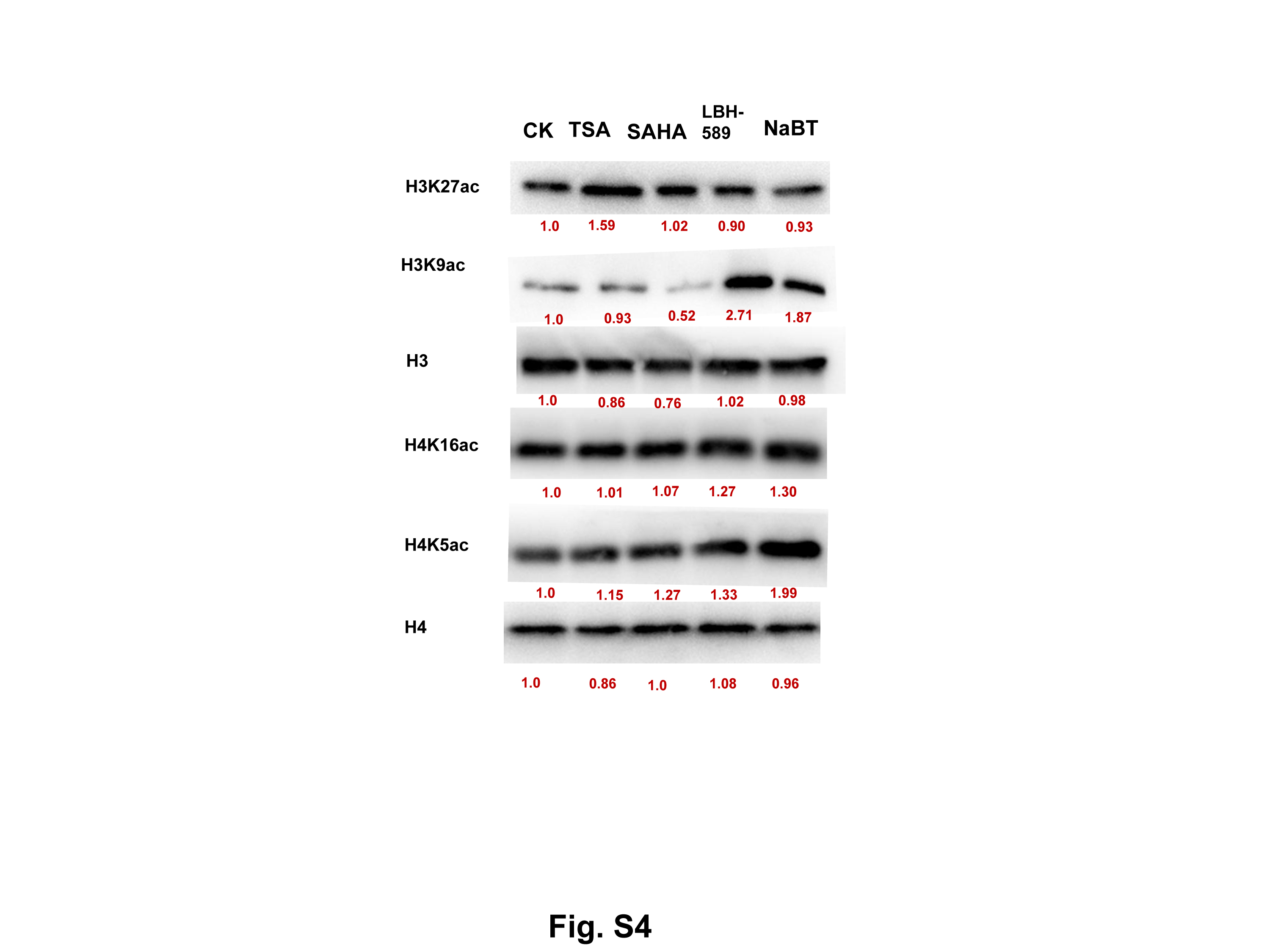

Supplement: Supplementary Figure 1 — The process of NaBT treatment and sample collection in this study. [file DataSheet_1.zip › Fig S4.jpg]

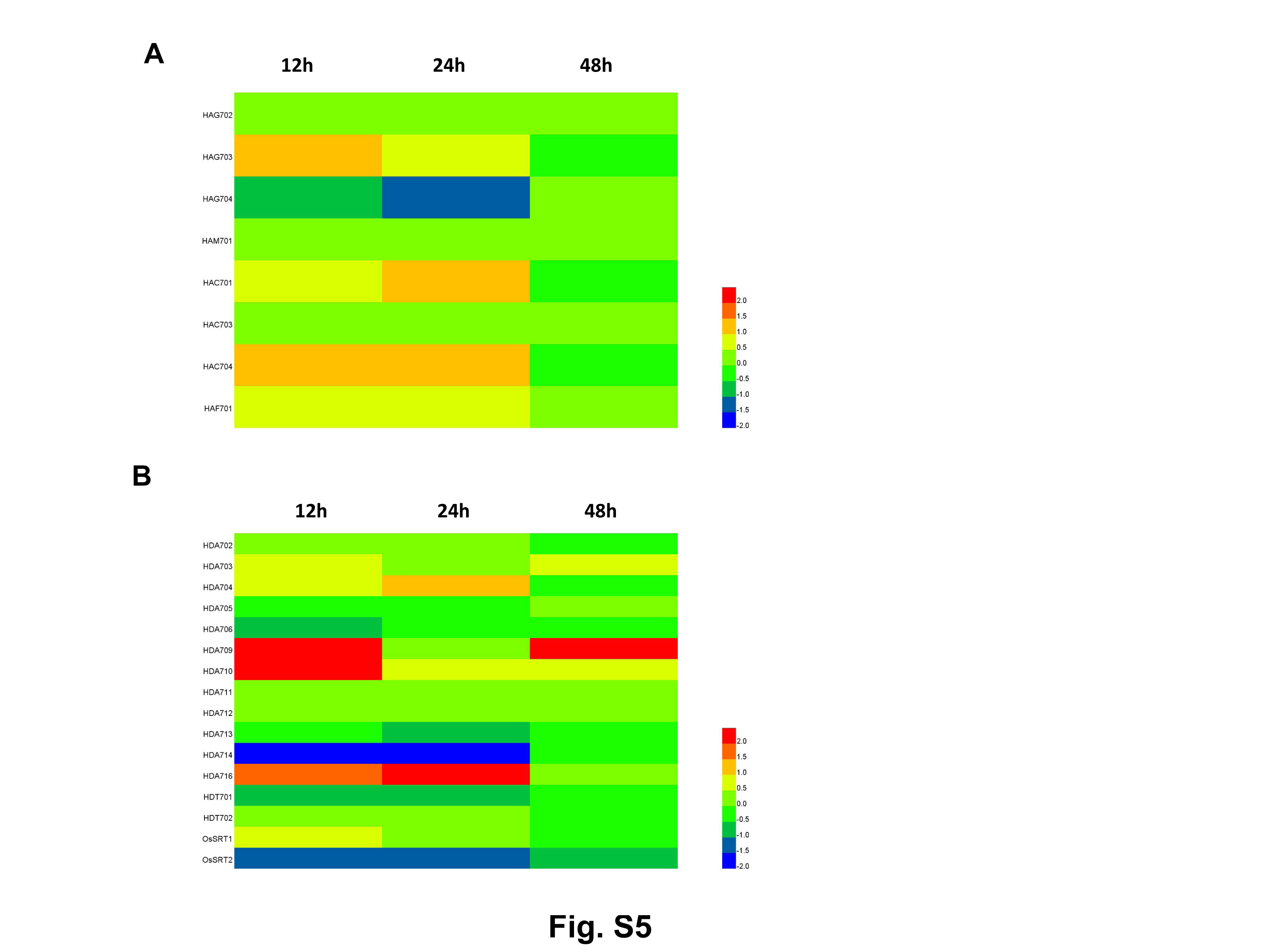

Supplement: Supplementary Figure 1 — The process of NaBT treatment and sample collection in this study. [file DataSheet_1.zip › Fig S5.jpg]

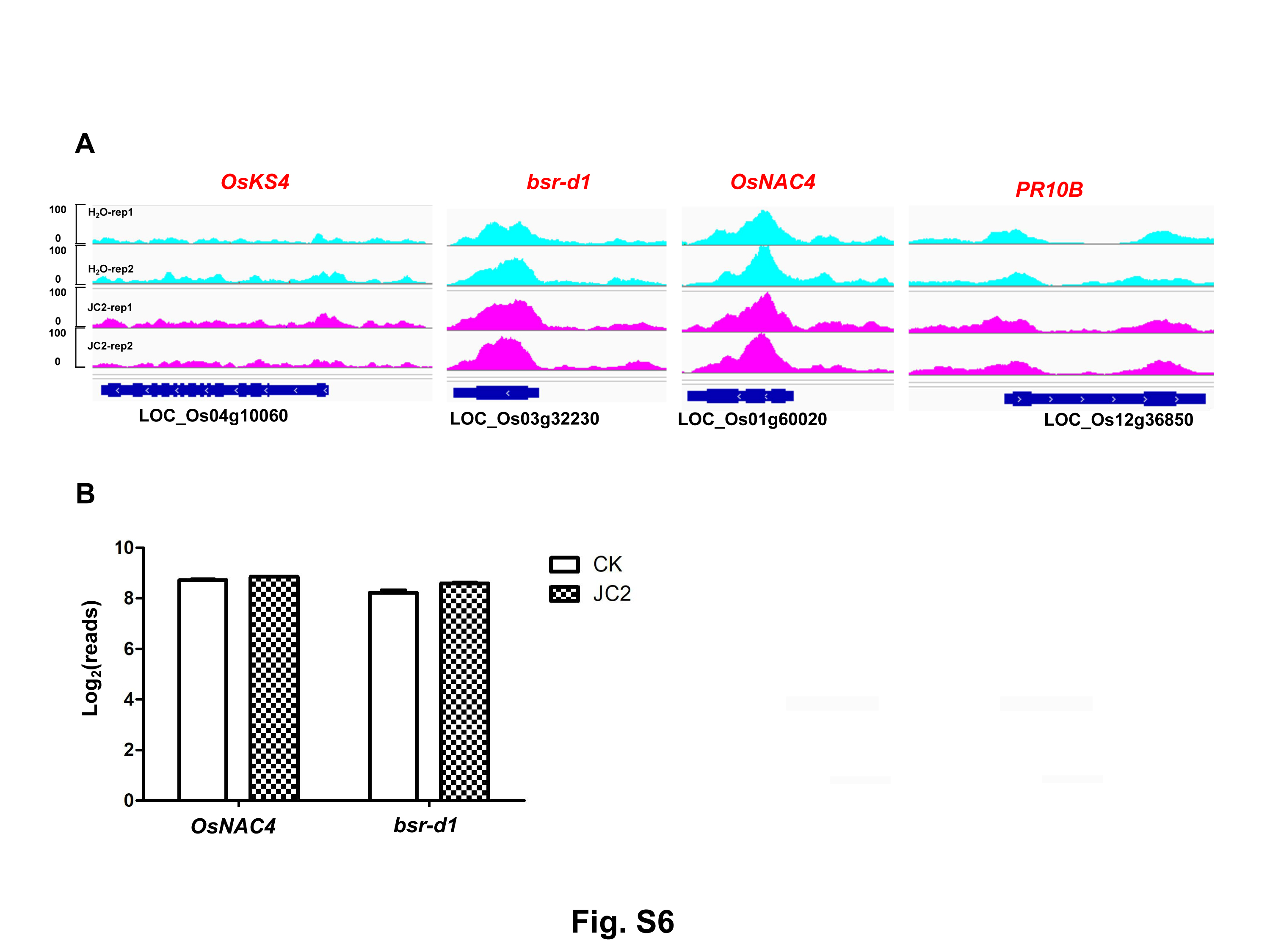

Supplement: Supplementary Figure 1 — The process of NaBT treatment and sample collection in this study. [file DataSheet_1.zip › Fig S6.jpg]

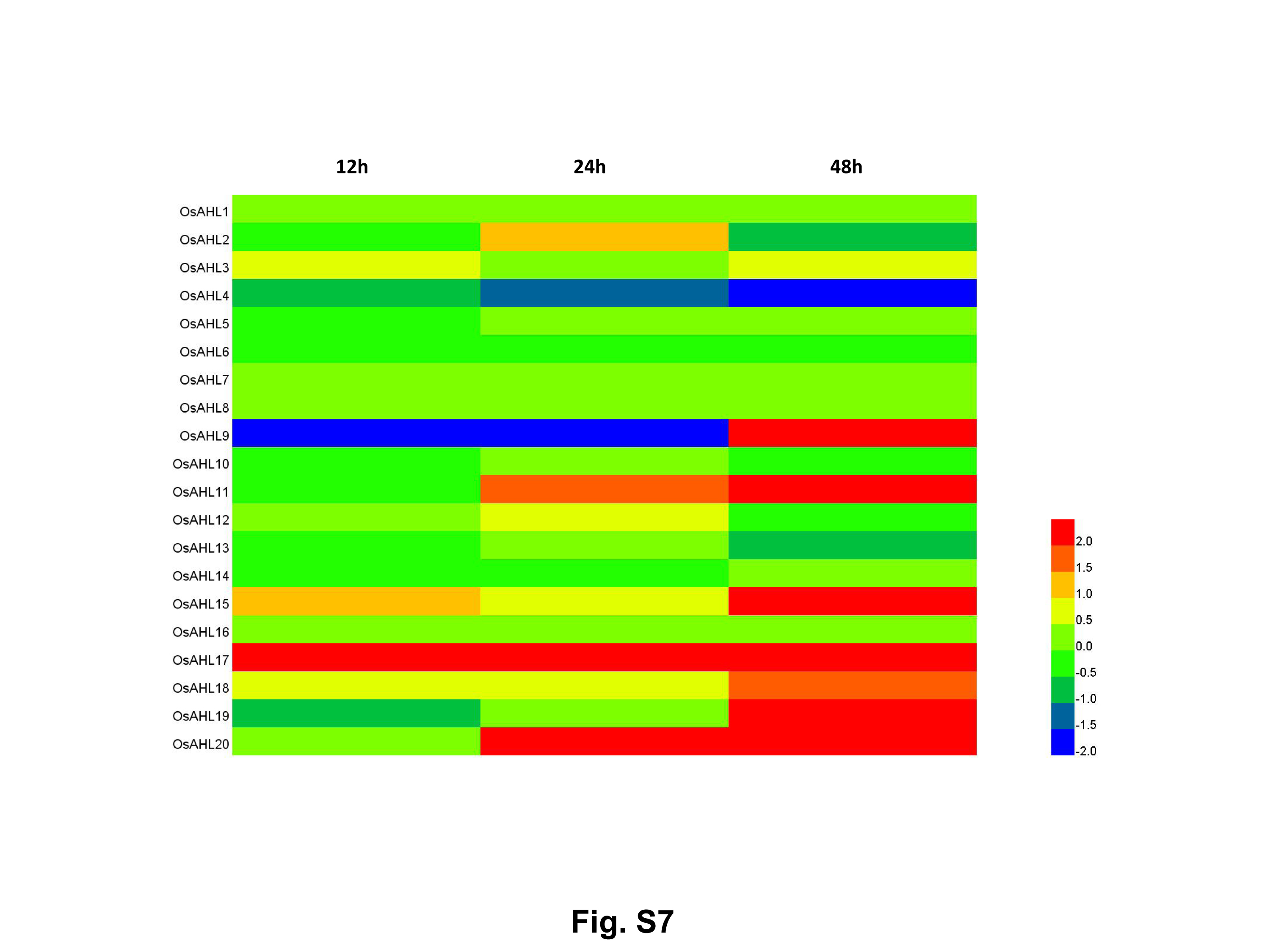

Supplement: Supplementary Figure 1 — The process of NaBT treatment and sample collection in this study. [file DataSheet_1.zip › Fig S7.jpg]
